# Hi-C profiling of cancer spheroids identifies 3D-growth-specific chromatin interactions in breast cancer endocrine resistance

**DOI:** 10.1186/s13148-021-01167-6

**Published:** 2021-09-17

**Authors:** Jingwei Li, Kun Fang, Lavanya Choppavarapu, Ke Yang, Yini Yang, Junbai Wang, Ruifeng Cao, Ismail Jatoi, Victor X. Jin

**Affiliations:** 1 Department of Gastrointestinal Surgery, The Third Xiangya Hospital, Central South University, Changsha, 410006 Hunan People’s Republic of China; 2 Department of Molecular Medicine, University of Texas Health San Antonio, San Antonio, TX 78229 USA; 3 Program of Biomedical Engineering, UTHSA-UTSA Joint Graduate Program, San Antonio, TX 78229 USA; 4 Department of Neurology, Xiangya Hospital, Central South University, Changsha, 410008 Hunan People’s Republic of China; 5 Minimally Invasive Surgical Center, Second Xiangya Hospital, Central South University, Changsha, 410011 Hunan People’s Republic of China; 6 Department of Pathology, Oslo University Hospital – Norwegian Radium Hospital, 0310 Montebello, Oslo, Norway; 7 Department of Biomedical Sciences, University of Minnesota Medical School, Duluth, MN 55812 USA; 8 Department of Surgery, University of Texas Health San Antonio, San Antonio, TX 78229 USA

**Keywords:** Hi-C, Looping genes, 3D spheroids, Organoids, Tamoxifen-resistant breast cancer

## Abstract

**Background:**

Organoids or spheroids have emerged as a physiologically relevant in vitro preclinical model to study patient-specific diseases. A recent study used spheroids of MCF10 cells to model breast cancer progression and identified targetable alterations more similar to those in vivo. Thus, it is practical and essential to explore and characterize the spheroids of the commonly used human breast cancer (BC) cells.

**Methods:**

In this study, we conducted Hi-C analyses in three-dimensional (3D) spheroids of MCF10A, MCF7 and MCF7TR cells and compared TADs and looping genes with those in 2D monolayers. Furthermore, we performed in silico functional analysis on 3D-growth-specific looping genes and to compare patient outcomes with or without endocrinal therapy. Finally, we performed 3C/RT-qPCR validations in 3D spheroids and 3D-FISH confirmations in organoids of breast cancer patient tissues.

**Results:**

We found that chromatin structures have experienced drastic changes during the 3D culture growth of BC cells although there is not much change in the quantity of chromatin domains. We also observed that the strengths of looping genes were statistically different between 2D monolayers and 3D spheroids. We further identified novel 3D growth-specific looping genes within Hippo relevant pathways, of which two genes showed potential prognostic values in measuring the outcome of the endocrine treatment. We finally confirmed a few selected genes in Hippo relevant pathways with enhanced looping in organoids of breast cancer patient tissues.

**Conclusions:**

Hence, our work has provided significant insights into our understanding of 3D-growth-specific chromatin architecture in tamoxifen-resistant breast cancer. Our analyses suggest that the strengthened looping-mediated Hippo relevant pathways may contribute to endocrine therapy resistance in breast cancer patients.

**Supplementary Information:**

The online version contains supplementary material available at 10.1186/s13148-021-01167-6.

## Background

Breast cancer (BC) is a heterogeneous disease that can be classified into many distinct subtypes based on its molecular, genetic and pathological characteristics [1]. Among them, 70% of BC patients have hormone-dependent estrogen receptor α (ERα or ER) positive tumors defined as luminal A and B subtypes [2–4], and estrogen (E2) plays a major role in the tumor initiation and progression [5–7]. Such subtypes of patients usually go through standard anti-hormone therapeutic treatment including tamoxifen, the most-prescribed selective estrogen receptor modulator (SERM) approved by the U.S. Food and Drug Administration (FDA) [8–11], or treatment with aromatase inhibitors. However, 30–50% of the patients who initially respond to tamoxifen develop de novo or acquired resistance [12]. Current two model systems routinely used to study underlying mechanisms and new treatments of BC are two-dimensional (2D) immortalized monolayer cell lines [13, 14] and patient-derived xenografts (PDX) [15, 16]. Despite offering many advantages and insight into the etiology of breast cancers, both models have some disadvantages [17]. For instance, derivation of PDXs is inefficient, labor-intensive and time-consuming while cell lines are unable to recapitulate the genetic heterogeneity and histopathological features of the individual patient’s tumor. Thus, both cancer models are limited in their translational applications and individualized therapeutic treatment on a broad scale. Due to these limitations, new or improved BC model systems should be utilized.

Organoids have first emerged as a physiologically relevant in vitro preclinical model to study stem cells, organ development and function, and patient-specific diseases [18–20]. Cancer organoids are subsequently established from individual-patient-derived tumor tissues embedded into a Matrigel (three-dimensional (3D) matrix) growing with high efficiencies into self-organizing organotypic structures [21, 22]. Many types of cancer organoids including BC organoids are now available as living biobanks of cancer organoids [23–26]. Sachs et al. has shown that a biobank of more than 100 BC organoids not only represents all major BC subtypes [27], but also retains expression of the BC biomarkers ER, progesterone receptor (PR) and epidermal growth factor receptor 2 (EGFR2 or HER2) and preserves histopathological and genetic features. Despite this available resource of BC organoids of primary tissues, they are not yet as abundant or easily accessible as 2D monolayers.

A recent study used 3D spheroids of MCF10 cells to model breast cancer progression and identified targetable alterations in conditions more similar to those encountered in vivo [28]. Other studies have used 42 different methods to establish 3D spheroids of breast cancer cells [29] and illustrated spheroids specific structure, growth and proliferation characteristics [30] and demonstrated they were associated with superior EMT and high resistance to the toxicological response compared with the standard 2D monolayer cultures. However, there lacks studies in understanding 3D chromatin architectures in BC spheroids or organoids. Thus, it is imperative to perform genome-wide Hi-C analysis in breast cancer spheroids.

In this study, we follow the reported protocols [18, 19] to establish 3D spheroids of three breast normal and cancer cells, MCF10A, MCF7 and MCF7TR. We then conduct in situ Hi-C analysis in 3D spheroids and compare chromatin interactions with those in 2D monolayers. Furthermore, we perform in silico functional analysis to identify novel signaling pathways associated with 3D-growth-specific looping genes and to compare patient outcomes with or without endocrinal therapy. Finally, we perform 3C/RT-qPCR validations in 3D spheroids and 3D-FISH confirmations in organoids of breast cancer patient tissues. To the best of our knowledge, this is the first study to interrogate the 3D chromatin landscape in BC spheroids.

## Methods

### 2D monolayers (cells) and 3D spheroids culture

MCF7 cells were grown in RPMI-1640 supplemented with 10% fetal bovine serum and 1% penicillin and streptomycin (pen/strep) until 90% confluent. MCF7TR cells were derived from Osborne et al. [31] and cultured in phenol red free RPMI-1640 containing 10% charcoal-stripped FBS, 1% pen/strep and 100 nM Tamoxifen (Sigma-Aldrich). Tamoxifen was replaced every 48 h. MCF10A cells were cultured in DMEM/F12 with 10% FBS and 1% pen/strep. All the cells were grown at 37 °C and 5% CO2 until they reach 90% confluence. Spheroids were generated as described in [18, 19]. Spheroids formed by MCF10A, MCF7 and MCF7TR were grown on Matrigel (Corning). Briefly, each plate was coated with 300ul of Matrigel and kept at 37 °C incubator for 30 min for gel formation. The Matrigel was overlaid with 2 ml of cell culture medium containing 5 × 10^4^ MCF10A, MCF7 or MCF7TR cells and incubated at 37 °C, 5% CO2 for 3–4 days to allow spheroid formation. The growth of spheroid was monitored everyday under a microscope and spheroids were split once they reached 80% confluence. We used 5–6 passages for MCF10A, MCF7 and MCF7TR cells to culture 3D spheroids.

### Immunostaining

Immunohistochemistry (IHC) staining was performed to detect against ER in 3D spheroids or organoids of MCF10A, MCF7 and MCF7TR. The arrays were sectioned in 3 µm thickness and placed on a poly-lysine coated slides to dry. The sections were dewaxed by baking the slides at 60 °C for 30 min followed by two washes of xylene, 5 min each at room temperature. Antigen Retrieval solution was performed by microwaving for 24 min in 10 mM sodium citrate solution [pH 6.0] and 30 min cooling to room temperature. Sections were submitted to endogenous peroxidase activity blocking with 3% hydrogen peroxidase for 20 min and rinsed with PBS for 9 min. Sections were blocked with 10% normal goat serum for 1 h at room temperature, then incubated with primary antibody ER [1:200; # MA5-14501; Invitrogen, US] for overnight in cold room. Spheroids were washed with PBS 1X three times and incubated with secondary Goat anti-rabbit Poly-HRP antibody [1:50; # 32260; Invitrogen, US] for 30–45 min. Diaminobenzidine (DAB)-based detection was performed to detect antibody binding and slides were counterstained with hematoxylin. Appropriate controls were used for all conditions.

### Western blotting

MCF10A, MCF7 and MCF7TR cells, spheroids or organoids were collected. Cells and spheroids were lysed in RIPA lysis buffer for 30 min on ice and centrifuged at 15000*g* for 15 min at 4 °C. Protein concentrations were measured using a Pierce BCA protein assay kit (Thermo Scientific). Protein samples (20–50 μg in different experiments) were separated by 10% Bis-Tris Plus gel (Invitrogen) by electrophoresis and then transferred to a nitrocellulose membrane. The membranes were blocked with 5% Bovine serum albumin (BSA) (Fischer scientific) in tris-buffered saline, 0.05% Tween 20 (TBST) at room temperature for 2 h. Membranes were then incubated with the primary antibody ERα and PR (1:1000) at 4 °C overnight. Following six minutes washing for four times in TBST, membranes were then incubated with HRP-conjugated secondary antibody (Invitrogen) (1:10,000) for 2 h at room temperature. After washing the membranes with TBST for four times six minutes each, proteins were detected using peroxidase detection reagent kit (Thermo scientific). Images were captured on CL-XPosure Film using Mini-Med 90 X-ray Film Processor. β-actin was used as an internal control. Antibodies used: 1) ER (Invitrogen, MA5-14501), HER2 (Invitrogen, MA5-14509), Ki67 (Invitrogen, MA5-14520), PR (Invitrogen, MA5-14505), β-actin (Abcam, ab227387), Rabbit Poly-HRP (Invitrogen, 32260). Image J software (U.S. National Institutes of Health, Bethesda, MD, USA) was used to quantify the bands on the membranes.

### Organoids of human breast tissues

Human primary breast tumor tissues were procured from Origene (OriGene Technologies Inc., Rockville, Maryland), and tamoxifen-treated recurrent breast tumor tissues were obtained from Ontario Tumor Bank. The use of human materials has been reviewed by UTHSA’s institutional review board. Organoids were grown as previously described in [18, 19]. Briefly, tissues were minced and then placed into a 50 ml conical tube containing 10 ml of washing medium AdDF+++ (Advanced DMEM/F12 containing 1 × Glutamax, 10 mM HEPES, and 1% Penicillin). After washing, tissues were transferred into the digestion medium containing AdDF++ and 0.1 mg/ml collagenase/Hyaluronidase (Stemcell Technologies 07912). Tubes containing minced tissue and collagenase were wrapped with a parafilm and incubated at 37 °C overnight on an orbital shaker. Next day, the mixture was sheared with 10 ml sterile pipet for 10 to 20 times. After digestion, 30 ml of AdDF+++ and 2% FBS were added to the mixture. The mixture was strained over 100um filter in to a new 50 ml tube and centrifuged at 4 °C, 600*g* for 5 min. The supernatant was discarded, and the organoids were pelleted. Additional mechanical shearing was done by adding 10 ml AdDF+++ and performed sequentially pipetting before the final pellet of breast organoids was obtained. The cell pellet was resuspended in matrigel and 50µL drop of Matrigel-cell suspension was seeded in the center of a well in a pre-warmed 24-well plate (1 drop/well). Avoid formation of air bubbles. Incubated the 24 well plate 1 h at 37 °C (until the matrix is solidified). Once the matrix is solidified, add 500µL of human breast organoid medium to the well. Incubate the culture under standard tissue culture conditions (37 °C, 5% CO2). Organoid culture medium was changed every 3–4 days, and organoids were passaged (between No. 2–6) using TrypLE Express (Invitrogen, 12605036) approximately every 2–4 weeks.

### In situ Hi-C

Hi-C was performed as previously described in [32, 33]. In brief, spheroids were cross-linked with 1% formaldehyde and lysed with cold lysis buffer to collect nuclei. The pelleted nuclei were digested with 200 units of HindIII (NEB, R3104L) at 37 °C for overnight. The HindIII digested fragment overhangs were filled with biotin-labeled dATP (Life Technologies, 19524-016) in a Klenow end-filling reaction. Four hundred units of T4 DNA Ligase (NEB, M0202) was added for ligation and samples were incubated for 4 h at room temperature with slow rotation. The ligation products were purified, and the chromatin was sheared to a size of 300–500 bp using Covaris sonicator (Covaris Woburn, MA). Dynabeads MyOne Streptavidin T1 beads (Life technologies, 65601) were used to pull down the biotin-labeled DNA. The end repair, dA tailing was performed and ligated with Illumina TruSeq adapters to form final Hi-C ligation products. Each Hi-C library was amplified with 12 cycles of PCR using Illumina primers. The Hi-C library was purified and then sequenced with Illumina HiSeq3000. A summary of replicated Hi-C data in Additional file 1: Table S1.

### RNA-seq

Total RNA was extracted by ZYMO Research Quick-RNA MiniPrep kit from lysed 10 million of cells and spheroids in RNA lysis buffer, then removed most of gDNA with the spin-away filter. After that, the mixture of RNA was transferred with ethanol to Zymo-Spin IIICG column to remove trace DNA by DNase I on the column, then washed twice with RNA wash buffer followed by elution with 50 μl DNase/RNase-free water. RNA-seq library was prepared with NEBNext® Poly(A) mRNA Magnetic Isolation Module (NEB #E7490). The Oligo dT Beads were washed with RNA-binding buffer and incubated with total 1 μg RNA to purify mRNA, followed with more washing by beads washing buffer. Then mRNA was eluted with elution buffer and reverse transcribed. After that, the first and the second strand cDNA were synthesized. After purification of Double-stranded DNA, adaptor was added. Adaptor-ligated DNA was enriched by PCR followed by purification, then the DNA library was sequenced with Illumina HiSeq3000. A summary of triplicated RNA-seq data in Additional file 1: Table S2.

### 3C-qPCR

3C-qPCR was performed as previously described in [32, 33]. The spheroids of MCF7 and MCF7TR were cross-linked with 1% formaldehyde for 10 min at room temperature. The reaction was quenched by the addition of 1 M glycine for 5 min at room temperature. The spheroids were lysed with 500 μL of cold lysis buffer (10 Mm Tris–HCl Ph 8.0, 10 Mm NaCl, 0.2% Igepal CA630) with protease inhibitor for at least 15 min on ice. After lysis, the cell nuclei were pelleted and the chromatin was digested using 200 units of HindIII (NEB) at 37 °C overnight and then the digestion was stopped at 65 °C for 20 min. Digested DNA fragments were ligated using T4 DNA ligase (NEB) for 4 h at 16 °C. Samples were reverse cross-linked with Proteinase K at 65 °C overnight. 3C samples were then purified using phenol–chloroform extraction. The 3C template was dissolved in 10 mM Tris–HCl and DNA concentrations were measured using Nanodrop. 3C primers are designed for a restriction fragment of interest. All pairs of primers amplified ligation products that were the result of head-to-head ligation of the corresponding restriction fragments. All 3C primers were designed by “Primer 3”. Primers used were listed in Additional file 1: Table S3. Interactions were measured using a 3c-qPCR assay for ligation products between each anchor HindIII fragment and each target HindIII fragment. The values that were obtained were normalized using GAPDH loading control.

### RT-qPCR

The spheroids of MCF7 and MCF7TR were treated and harvested with Cell Recovery Solution (Corning Incorporated, Corning, NY, USA) to remove the Matrigel. Total RNA was extracted from spheroids using Quick -RNA^TM^ Mini Prep (Zymo Research, USA) according to the manufacturer's instructions. Each 10 μl reaction consist of SuperScript III RT/ Platinum Taq mix, 5ul of 2X SYBR green reaction mix, 1ul of test primer, 300 ng RNA and distilled water. Quantitative Real-time PCR was performed on Light Cycler® 480 Instrument II real-time PCR system (Roche Diagnostics, Penzberg, Germany) and Ct values were outputted for quantification. Initial enzyme activation was performed at 95 °C for 15 min, followed by 70 cycles of denaturation at 95 °C for 15 s and primer annealing/extension at 60 °C for 70 s. Melting curve analysis was performed at 95 °C for 5 s, 65 °C for 60 s and 45 °C for 30 s. Primers used were listed in Additional file 1: Table S4. The expression levels of target genes were normalized against endogenous control ACTB. Data analysis was doing using 2-ΔΔCt method. Each PCR reaction was performed in triplicate, and the data presented were the average of three independent experiment results for all PCR reactions.

### 3D-FISH

3D-FISH was performed to determine the separation of the promoter-distal loop of a gene as described in [34]. Fosmid- and BAC-DNA constructs were received as bacterial stab received from BACPAC resource to be streaked out onto agar plates with 12.5 µg/ml of Chloramphenicol. Growing BAC/Fosmid clones overnight at 37 °C in shaker incubator. Isolate DNA using Nucleobond purification MAXI PREP kit (TaKaRa, 740579) and store at 4 °C until further use. DNA-FISH probe labeling was performed using the BioPrime DNA Labeling System (ThermoFisher, 18094011), which consists in the direct incorporation of fluorescently labeled dUTP by DNA extension using Klenow fragment and random primers. After purification and precipitation, store labeled probes at -20 °C in the dark. Simultaneous DNA denaturation and hybridization was performed in prepared spheroids of MCF7/MCF7TR and PT/RT organoids, respectively. After overnight hybridization, coverslips were then sealed onto the glass slide for microscopy observation. To determine inter-probe distances (promoter-enhancer), 3D images from 50 nuclei were analyzed using NEMO (https://forge-dga.jouy.inra.fr/projects/nemo). Probes used were listed in Additional file 1: Table S5.

### Differential TADs or TAD changes

We used HiC-Pro [35] to process the raw Hi-C data to get the iced contact matrix. Since we used some of Hi-C data from previous studies, we firstly examine if there is any batch effect existed. We found that all data didn’t have any batch effect except MCF10A_2D from Barutcu et al. [36] (Additional file 1: Figs. S1–S2). We then used a Hi-C matrix based LOWESS normalization method to identify and filter out the biases in MCF10A_2D data based on the MCF7_2D from that same study (Additional file 1: Method and Figs. S3–S4). We used TopDom [37] to call topological associated domains (TADs) with the default parameters. We also used a down-sampled MCF7TR_3D data to confirm that there was no effect of the variability of the number of valid pairs on the TADs calling process (Additional file 1: Fig. S5). We then compared the TAD changes between 2D monolayers and 3D spheroids by using TADs in 2D monolayers as a control. We defined eight types of changes as our previous study [33]: No change: no changed TAD size and length between two conditions, A and B; b) Conserve-expand: a TAD identified in both conditions A and B, with the length of the TAD increasing by at most 300 Kb in condition B; c) Conserve-shrink: a TAD identified in both conditions A and B, with the length of the TAD decreasing by at most 300 Kb in condition B; d) Shift: a TAD identified in condition B that overlaps with a TAD in condition A, with the position shifting by more than 300 Kb; e) Split: a TAD in condition A becoming multiple TADs in condition B; f) Fuse: multiple TADs in condition A becoming one TAD in condition B; g) Neo: a border boundary or GAP in condition A becoming a TAD in condition B; h) Del: a TAD in condition A becoming a GAP or border boundary in condition B. Types a-c) are relatively conserved (RC) and types d-h) are drastically changed (DC). This visualization of the TAD was accomplished by HiC-explorer [38].

### P1D1 loops, looping genes and strengthened looping genes

We used HiSIF [39] to identify significant interaction fragments (SIFs) with all valid pairs from HiC-Pro [35] and HISIF parameters of t = 1, s = 2, *p* = 1 29, w = 50, 500, 20,000. We further used FDR ≤ 0.1 as the cutoff to select the final set of SIFs. We defined a P1D1 loop or a loop as if one end of the SIF on the promoter region of an annotated protein-coding gene and the other end on the distal region of the same gene. The assigning priority is to assign a SIF to the promoter region before the distal region. For instance, if an end of a SIF covers both the distal region of one gene and the promoter region of another gene, we assign this end as the promoter region of the gene. The promoter region of a gene was defined as from 4 Kb upstream of 5’TSS to 1 Kb downstream of 5’TSS while the distal region of a gene was defined as 100–10 Kb upstream of 5’TSS and 10–100 Kb downstream of 5’TSS. We defined a gene with at least one loop as a looping gene.

To identify the differential or strengthened looping genes (SLGs) between two conditions, we first defined a Valid Pairs Per Million (VPPM) to measure the strength of the loop from the output of HISIF within each P1D1 loop as $Strength \,of\, the\, loop= \frac{Valid \,pairs \,within \,a \,loop}{Total \,valid \,pairs }*{10}^{6}.$ We summed all VPPMs of one or more loops associated with the same gene to a single VPPM to represent that gene. To obtain a strengthened looping gene (SLG) in MCF7TR_3D compared to MCF7_3D, we subtracted the VPPMs in MCF7_3D from the VPPM in MCF7TR_3D and normalized all VPPMs values into the range of 0–1.0 by z-score. We empirically set the cutoff of ≥ 0.15 as strengthened loop genes in MCF7TR_3D. With this definition, we only consider strengthened loop genes in one condition as a differential looping gene (DLG). We thus obtained two different sets of DLGs in MCF7TR_3D and MCF7_3D, respectively.

### Differentially expressed looping genes (DELGs)

RNA-seq data were first performed quality control by Trim Galore [40] and mapped to hg19 by Hisat2 [41] with its default parameters. The read counts for each gene were calculated by featureCounts with parameters -s 2 and -M. Differentially expressed genes (DEGs) were identified by DESeq2 [42] with the cutoffs as abs(log2FoldChange) ≥ 0.58 and p-value ≤ 0.05. The overlapping genes between strengthened looping genes (SLGs) and DEGs were thus considered as differentially expressed looping genes (DELGs).

### Gene ontologies (GO), pathways and patients survival analyses

We used Enrichr [43] to perform the pathway analysis on DELGs in MCF7TR_3D. This tool has collected data libraries for transcriptional regulation, pathways and protein interactions, ontologies including GO and the human and mouse phenotype ontologies, signatures from cells treated with drugs, and expression of genes in different cells and tissues. We also used an online survival tool (www.kmplot.com) [44] to rapidly assess the effect of genes on breast cancer prognosis using microarray data of 1809 patients. In order to analyze the prognostic value of a particular gene, the cohorts were divided into two groups (low and high expression level) according to the median expression of the gene. The two groups can be compared in terms of relapse free survival, overall survival and distant metastasis free survival. A survival curve was displayed with the hazard ratio with 95% confidence intervals and a logrank p value.

## Results

### Establishing the 3D spheroids of breast normal and cancer cells

**Fig. 1:**
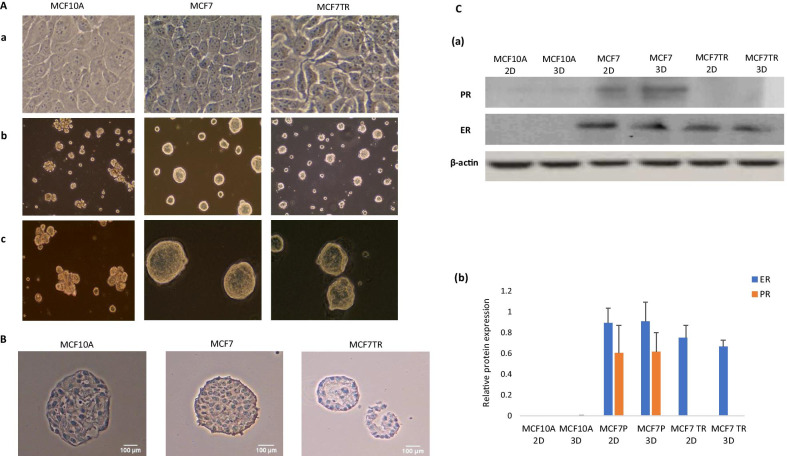
Establishing 3D spheroids of breast normal and cancer cells. **A** Phase contrast images of 2D cells of MCF10A, MCF7 and MCF7TR at 20X magnification (**a**), of 3D spheroids at 10X magnification (**b**) and of 3D spheroids at 20X magnification (**c**), respectively. **B** IHC staining showing higher ER in 3D spheroids of MCF7 (MCF7_3D) and in 3D spheroids of MCF7TR (MCF7TR_3D) but absent in 3D spheroids of MCF10A (MCF10A_3D) with an imaging resolution of 100 µm or 40X magnification. **C** Western blotting in gel images (**a**) and in histograms (**b**) showing both ER and PR proteins were highly expressed in MCF7_2D/3D, only ER was highly expressed in MCF7TR_2D/3D, neither proteins was explicitly expressed in MCF10A_2D/3D. β-actin levels were measured as a loading control. All data in (**b**) corresponded to the mean ± SD of three independent experiments

To comprehensively examine genome-wide chromatin interactions in 3D spheroids, we first followed the reported protocols [18, 19] to establish 3D spheroids of MCF10A, MCF7 and MCF7TR cells (Fig. 1A). We confirmed the expression of ER in 3D spheroids of MCF7 (MCF7_3D) and MCF7TR (MCF7TR_3D) respectively, but not in 3D spheroids of MCF10A (MCF10A_3D) by IHC staining (Fig. 1B). We further examined ER and PR protein expression in 3D spheroids as well as in the corresponding 2D monolayers, MCF10A_2D, MCF7_2D and MCF7TR_2D. We observed that ER protein expression was higher in both MCF7_2D/3D and MCFTR_2D/3D. However, we found that PR protein expression was higher in MCF7_2D/3D, but unexpressed in MCF7TR_2D/3D. Neither ER nor PR protein expression was detected in MCF10A_2D/3D (Fig. 1C). Taken together, our data demonstrated that we had successfully established 3D spheroids of MCF10A, MCF7 and MCF7TR cells, respectively.

### Identifying the differential TADs between 2D monolayers and 3D spheroids

**Fig. 2:**
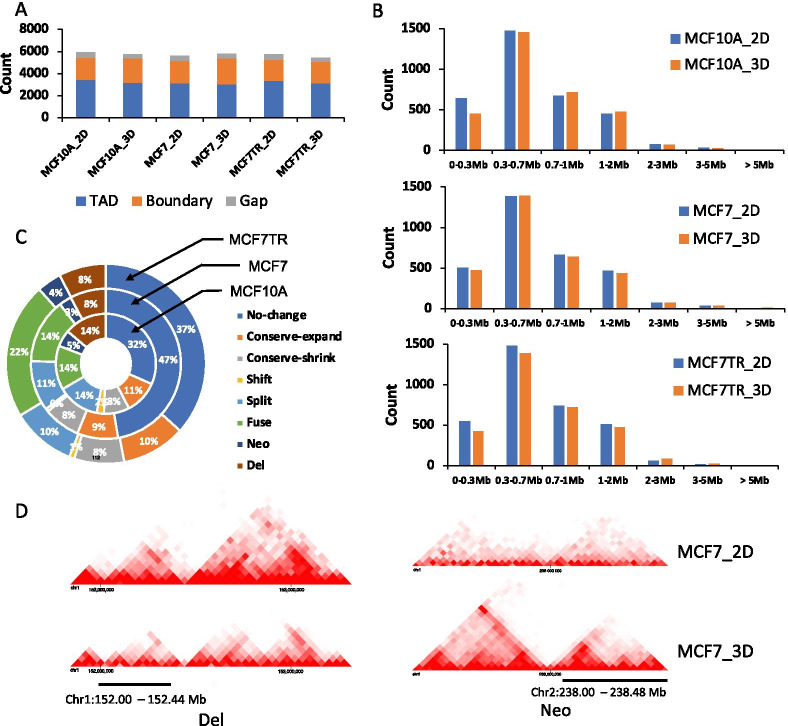
Identifying the differential TADs between 2D monolayers and 3D spheroids. **A** Bar graph depicting the number of the TADs, Boundaries, and Gaps were identified for MCF10A_2/3D, MCF7_2D/3D and MCF7TR_2D/3D, respectively. **B** The distribution of TAD size showing there is no significant difference between MCF10A_2D/3D, MCF7_2D/3D and MF7TR_2D/3D, respectively. **C** Doughnut chart showing a percentage breakdown for TAD changes between 2D and 3D conditions for three cell types respectively. **D** Screenshots demonstrating two types of TAD change, Del and Neo. Region Chr1: 152.00–152.44 Mb (left panel) and Chr2:238.00–238.48 Mb (right panel)

We have performed in situ Hi-C in the newly established 3D spheroids, MCF10A_3D, MCF7_3D and MCF7TR_3D, each with biological replicates (Additional file 1: Table S1, Fig. S1). We then applied TopDom [29] on the combined replicates to identify TADs, Gaps and Boundaries in 3D spheroids as well as in 2D monolayers from previous studies [32, 33]. We found that there were ~ 3000 TADs each for three 2D monolayers and three 3D spheroids, respectively, and the ratio of the number of Gaps/Boundaries to the number of TADs were very similar among all six 2D/3D culture conditions (Fig. 2A). We then examined the size of TADs and found a majority of their size were within 0.3–2 Mb for both 2D monolayers and 3D spheroids (Fig. 2B). Interestingly, the distribution of the number of different sizes of TADs was very similar among all six conditions. We then compared the changes of TADs between 2D monolayers and 3D spheroids using the eight types of TAD changes defined in our recent study [33]: a) No change; b) Conserve-expand; c) Conserve-shrink; d) Shift; e) Split; f) Fuse; g) Neo (from a border boundary or GAP to a new TAD); h) Del (from a TAD to GAP or border boundary). Remarkably, we observed that the relatively conserved (RC) TADs including No change, Conserve-expand and Conserve-shrink, were accounted for between 40 and 65% of all types of TAD changes for all three 2D/3D comparisons, and the drastically changed (DC) TADs including Shift, Split, Fuse, Neo and Del, were accounted from 35 to 60% (Fig. 2C). Surprisingly, we found very few Shift TADs between all three 2D/3D comparisons. Screenshots of two examples of TAD changes between MCF7_2D and MCF7_3D, Del and Neo are shown in Fig. 2D. Taken together, our analyses suggested that chromatin structures have experienced drastic changes during the 3D culture growth of BC cells although there isn’t much change in the quantity of chromatin domains.

### Identifying the differential looping genes between 2D monolayers and 3D spheroids

**Fig. 3:**
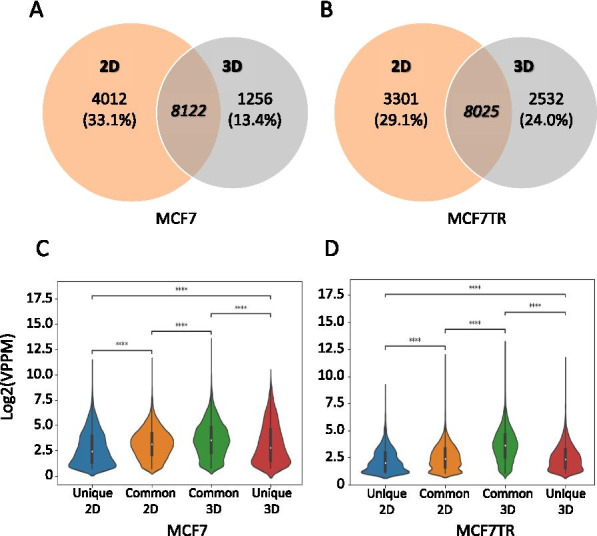
Identifying the differential looping genes between 2D monolayers and 3D spheroids. **A** Venn diagram showing common and unique P1D1 loops between MCF7_2D/3D. **B** Venn diagram showing common and unique P1D1 loops between MCF7TR_2D/3D. **C** Violin-plot showing the distributions of looping strengths of 2D unique, common and 3D unique looping genes in MCF7. **D** Violin-plot showing the distributions of looping strengths of 2D unique, common and 3D unique looping genes in MCF7TR

We applied HiSIF [39] to identify significant interaction fragments (SIFs) for the Hi-C data in both 2D monolayers and 3D spheroids. At the optimal parameters set (t = 1, 20 Kb, FDR = 0.1), we obtained a total of 577,585 for MCF7_2D, 431,056 for MCF7_3D, 327,064 for MCF7TR_2D and 319,644 for MCF7TR_3D, respectively. We further examined the promoter-centric SIFs or promoter-distal loops (P1D1 loops or loops), whereas a loop is defined in the following: one end of the SIF is within the promoter region of a protein coding gene (defined as upstream (—) 4 Kb to downstream (+) 1 Kb of transcription start site (5’TSS)) and the other end is within the non-promoter region of the same gene (defined as ± 100 Kb to ± 10 Kb of 5’TSS). We were able to identify 4012 unique in MCF7_2D, 1256 unique in MCF7_3D and 8122 common between MCF7_2D and MCF7_3D (Fig. 3A, Additional file 2: File S1); 3301 unique in MCF7TR_2D, 2532 unique in MCF7TR_3D and 8025 common between MCF7TR_2D and MCF7TR_3D (Fig. 3B, Additional file 2: File S1).

In order to measure the looping strength of a looping gene, we computed a VPPM (see “Methods” section) for each of loops within a looping gene and then added all VPPMs together as the looping strength. We then compared the looping strength of looping genes between 2D monolayers and 3D spheroids (Fig. 3C, D). As expected, for all three cell types, we found the distributions of the strengths of looping genes were statistically different between Unique_2D and Unique_3D, between Unique_2D and Common_2D as well as between Unique_3D and Common_3D, respectively. However, we surprisingly observed a statistical difference between Common_2D and Common_3D, suggesting that the same gene can exert different looping strength at the distinct culture conditions. Our analysis may also suggest VPPM could be an appropriate measure to examine the differentially looping genes between two experimental conditions.

### Characterizing 3D spheroid-specific looping genes

**Fig. 4:**
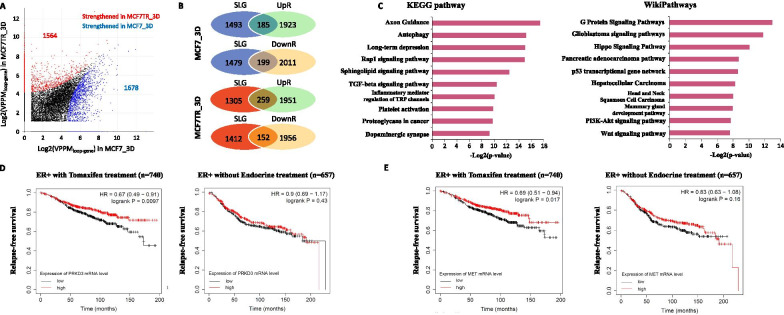
Characterizing 3D spheroid-specific looping genes. **A** 3D scatterplot showing identified 1678 and 1564 strengthened loop genes in MCF7_3D and MCF7TR_3D, respectively. **B** Venn diagrams showing 384 of 1678 strengthened looping genes in MCF7_3D as well as 411 of 1564 strengthened looping genes in MCF7TR_3D were differentially expressed between MCF7_3D vs MCF7TR_3D, respectively. **C** Bar graphs showing the top 10 enriched KEGG pathway and WikiPathway terms for the strengthened looping and differentially expressed genes in MCF7TR_3D. **D** Patients survival analysis of PRKD3 within a Hippo relevant pathway in ER + breast cancer patients with tamoxifen treatment and without endocrine treatment. Expression levels of PRKD3 gene are classified as low or high (black or red lines, respectively) based on the comparison of its median cut-off value. (**E**) Patients survival analysis of MET within a Hippo relevant pathway in ER + breast cancer patients with tamoxifen treatment and without endocrine treatment. Expression levels of MET gene are classified as low or high (black or red lines, respectively) based on the comparison of its median cut-off value

Next, we wanted to examine 3D spheroid-specific looping genes. We computed the difference of VPPMs between MCF7 and MCF7TR cells. We identified 1678 and 1564 strengthened looping genes (SLGs) in MCF7_3D and MCF7TR_3D respectively (Fig. 4A, Additional file 3: File S2). In order to examine the gene expression levels for those 3D spheroid-specific SLGs in MCF7_3D or MCF7TR_3D, we performed RNA-seq in both 2D monolayers and 3D spheroids for both cell types (Additional file 1: Table S2) and identified 4318 differentially expressed genes (DEGs) between MCF7_3D and MCF7TR_3D (Additional file 4: File S3). Remarkably, we found 384 of 1678 SLGs in MCF7_3D as well as 411 of 1564 SLGs in MCF7TR_3D showing differentially expressed between MCF7_3D vs MCF7TR_3D, respectively, in which these genes were considered as differentially expressed looping genes (DELGs) (Fig. 4B, Additional file 1: Fig. S6). We then performed Gene Ontology (GO) and Pathway analyses on MCF7TR_3D specific DELGs. Interestingly, we identified not only some known signaling pathways previously reported in tamoxifen-resistant breast cancer including Wnt signaling, TGF-beta and PI3K-Akt signaling [32, 33, 38, 45], but also a few novel 3D spheroid-specific pathways such as Hippo and Rap1 signaling pathways, as well as a upstream pathway regulating Hippo signaling: G Protein pathways (Fig. 4C). We further conducted patient survival analysis on looping genes within Hippo relevant pathways and Rap1 signaling pathways. We found that higher expression of PRKD3, a component gene in G Protein signaling, and higher expression of MET, a component gene in Rap1 signaling pathway, were associated with the worse relapse-free survival (RFS) in the tamoxifen-treated ERα + patients but not with those patients without any endocrine treatment (Fig. 4D, E). Our analysis reveals 3D-growth-specific looping-mediated signaling pathways in tamoxifen-resistant breast cancer cells and indicates a potential prognostic value of Hippo relevant pathways genes in measuring the outcome of the endocrine treatment.

### Examining 3D-growth-specific looping genes in organoids of breast cancer tissues

**Fig. 5:**
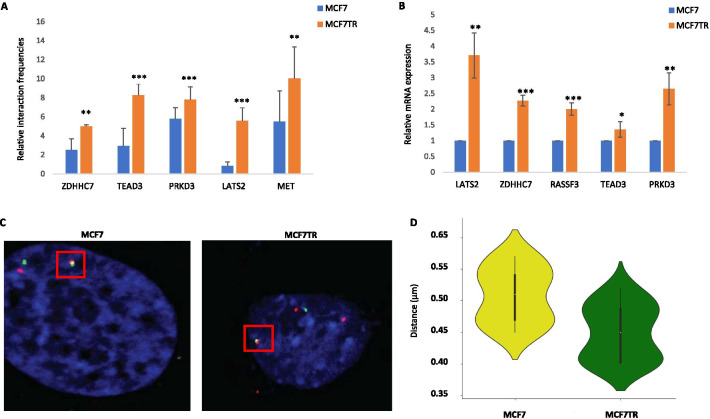
Conducting 3C/RT-qPCR and 3D-FISH validations of differentially expressed looping genes in 3D spheroids of BC cells. **A** 3C-qPCR showing enhanced interaction frequencies of each gene loop for ZDHHC7, TEAD3, PRKD3, LATS2 and MET in MCF7TR_3D compared to those in MCF7_3D. Three biological replicates were performed for each gene loop with a statistical significance (***p ≤ 0.01; **p ≤ 0.05) by one-tail paired t test analysis. Error bars represent standard deviation with three experiments. **B** RT-qPCR showing increased gene expression levels of ZDHHC7, TEAD3, PRKD3, LATS2 and RASSF3 in MCF7TR_3D compared to those in MCF7_3D. Three biological replicates were performed for each gene loop with a statistical significance (***p ≤ 0.01; **p ≤ 0.05; *p ≤ 0.1) by one-tail paired t test analysis. Error bars represent standard deviation with three experiments. **C** 3D-FISH images showing interaction frequency of the PRKD3 loop in MCF7TR_3D and MCF7_3D, respectively. BAC probe combinations: promoter (green) and distal region (red) *n* = 50, DAPI DNA stain (blue). Square boxes in red represent the magnified view of each interaction. Scale bar at 5 µm. **D** Distributions of measured distances of the PRKD3 loop are significantly different for MCF7TR_3D vs. MCF7_3D (*p ≤ 0.1) by one-tail paired t test analysis. Distances were measured between the closest two foci in each nucleus

To further examine 3D-growth-specific looping genes within Hippo relevant pathways, we first conducted 3C/RT-qPCR validations in 3D spheroids and were able to confirm a few selected genes that showed enhanced looping interaction and higher gene expression in MCF7TR_3D vs MCF7_3D (Fig. 5A, B, Additional file 1: Tables S3–S4). Remarkably, our 3D-FISH results further illustrated that the distribution of measured distances of the PRKD3 loop in MCF7TR_3D was significantly different from the distribution in MCF7_3D (Fig. 5C, D, Additional file 1: Table S5). Together, these two lines of distinct evidence confirmed the looping genes associated with Hippo relevant pathways indeed displayed 3D-growth-specific manner in the 3D culture BC model.

**Fig. 6:**
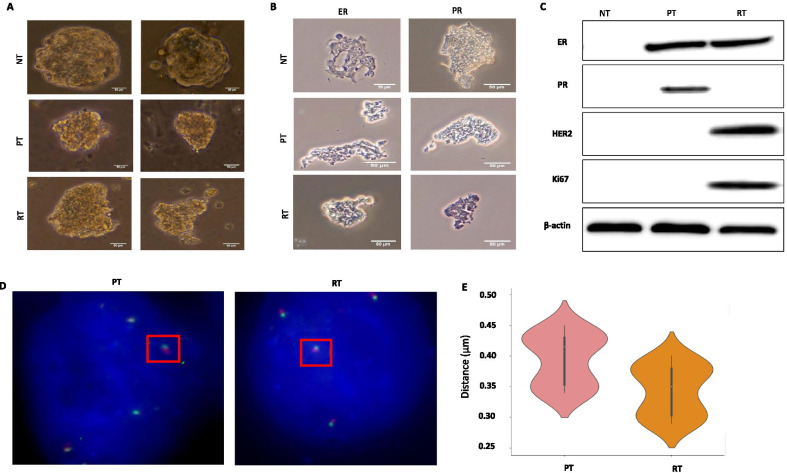
Conducting 3D-FISH validations of the PRKD3 loop in organoids of BC tissues. **A** Images of organoids derived from normal breast tissue (NT), primary breast cancer tissue (PT) and tamoxifen-treated breast cancer recurrent tissue (RT). Scale bar at 50 µm. **B** IHC staining of ER and PR in organoids of NT, PT and RT respectively with a higher magnification (40X) and a scale bar at 50 µm. **C** Western blotting showing ER, PR, HER2 and Ki67 protein levels in organoids of NT, PT and RT, respectively. β-actin levels were measured as a loading control. **D** 3D-FISH images showing interaction frequency of the PRKD3 loop in organoids of PT and RT, respectively. BAC probe combinations: promoter (green) and distal region (red) *n* = 50, DAPI DNA stain (blue). Square boxes in red represent the magnified view of each interaction. Scale bar at 5 µm. **E** Distributions of measured distances of the PRKD3 loop are significantly different for PT vs. RT (*p ≤ 0.1) by one-tail paired t test analysis. Distances were measured between the closest two foci in each nucleus

Organoids of BC tissues have just been established as a biobank resource [27]. How they can be practically used for examining chromatin looping remains elusive. We were able to establish three types of organoids from human tissues, breast normal tissue (NT), breast cancer primary tissue (PT) and tamoxifen-treated breast cancer recurrent tissue (RT) (Fig. 6A–C, Additional file 1: Fig. S7). We then conducted 3D-FISH validation of the PRKD3 loop in these newly established organoids, PT and RT. Clearly, we observed there was a higher interaction frequency of the PRKD3 loop in two organoids of PT than in two organoids of RT (Fig. 6D, E). Taken together, our results demonstrate that organoids of breast cancer tissues can recapitulate differential loop strengths identified in 3D spheroids, therefore could serve as a better preclinical breast cancer model for studying 3D chromatin regulation.

## Discussion

Despite numerous studies that have revealed the principles of chromatin architectures in normal and disease states [32, 46–49] and identified cancer-specific TADs and looping genes [32, 48, 49], very few studies were conducted in 3D-growth culture conditions including spheroids and organoids. Given that there are higher bio-similarities between organoids and in vivo cellular organ structure, it is imperative to perform Hi-C profiling in 3D spheroids or organoids to identify 3D-growth-specific chromatin interactions. In this study, we, for the first time, identified thousands of 3D-growth-specific TADs and looping genes in 3D spheroids of breast cancer cells (Figs. 2 and 3). Undoubtedly, our genome-wide chromatin interaction data provide a rich resource for further studying the mechanism of looping-mediated signaling pathways or genes in contributing to breast cancer endocrine resistance.

Interestingly, we identified novel 3D-growth-specific signaling pathways including Hippo and Rap1 signaling pathways, as well as G Protein signaling pathways, a upstream pathway regulating Hippo signaling (Fig. 4C). Furthermore, we found the gene expression of PRKD3 and MET within Hippo relevant pathways were able to stratify tamoxifen-treated ERα + patients into better and worse groups of relapse-free survival (RFS), respectively (Fig. 4D, E), supporting a notion that PRKD3 and MET may be used as predictive biomarkers for response to endocrine therapy in ERα + breast cancer patients. Although previous studies have demonstrated that PRKD3 is an oncogenic function in invasive breast cancer [50], and MET has a potential prognostic value in breast cancer [51], our study further elicits their potential prognostic values in predicting the outcome of endocrine therapy.

Remarkably, we confirmed the selected strengthened looping genes and differential expressed genes within Hippo relevant pathways in 3D spheroids (Fig. 5) and further validated PRKD3 in organoids of breast cancer tissues by 3D-FISH (Fig. 6). This result is highly significant as it might serve as a better in vitro preclinical model for further translational studies including drug screening, cancer modeling and toxicity testing. However, we recognize several limitations in this validation. For example, we only performed 3D-FISH on PRKD3 and thus need to expand to more genes within Hippo relevant pathways or other biological signaling pathways. In addition, it is necessary to re-examine these 3D-growth-specific looping genes in an in vivo mouse model of breast cancer.

## Conclusions

Collectively, our work has provided significant insights into our understanding of 3D-growth-specific chromatin architecture in tamoxifen-resistant breast cancer. Our analyses suggest that the strengthened looping-mediated Hippo relevant pathways may contribute to endocrine therapy resistance in breast cancer patients. The future of breast cancer research may benefit greatly from the additional scrutinization of the Hippo relevant pathways for the development of better prognostic biomarkers and the designing of patient selection for targeted endocrine treatment.

## Supplementary Information

**Additional file 1**. Supplementary information includes Supplemental Method, seven Supplemental figures, five Supplemental tables.

**Additional file 2**. Supplemental excel file for listing common and unique loop genes in MCF7_2D, MCF7_3D, MCF7TR_2D and MCF7TR_3D.

**Additional file 3**. Supplemental excel file for listing strengthened loop genes in MCF7_2D, MCF7_3D, MCF7TR_2D and MCF7TR_3D.

**Additional file 4**. Supplemental excel file for listing differential expressed genes between MCF7_3D. vs. MCF7TR_3D as well as MCF7_2D.vs.MCF7TR_2D.

## Data Availability

Raw and processed in situ Hi-C for 3D spheroids of MCF10A, MCF7, MCF7TR and Raw and processed RNA-seq data for 2D monolayers and 3D spheroids of MCF10A, MCF7 and MCF7TR are deposited in GEO under accession number GSE165572.

## Abbreviations

BC: Breast cancer
2D: Two dimensional
SERM: Selective estrogen receptor modulator
FDA: Food and Drug Administration
PR: Progesterone receptor
EGFR2 or HER2: Epidermal growth factor receptor 2
TAD: Topological associated domain
SLGs: Strengthened looping genes
DELGs: Differentially expressed looping genes
3D: Three dimensional
ERα or ER: Estrogen receptor α
PDX: Patient-derived xenografts
IHC: Immunohistochemistry
VPPM: Valid pairs per million
DLG: Differential looping gene
DEGs: Differentially expressed genes
