# Supplementary material for: Hi-C profiling of cancer spheroids identifies 3D-growth-specific chromatin interactions in breast cancer endocrine resistance
Source: Clin Epigenetics. 2021 Sep 17;13:175. doi: 10.1186/s13148-021-01167-6 (PMC8447690; doi:10.1186/s13148-021-01167-6)
Supplement: Supplementary file 1 — Additional file 1. Supplementary information includes Supplemental Method, seven Supplemental figures, five Supplemental tables. [file 13148_2021_1167_MOESM1_ESM.docx]

**Hi-C profiling of cancer spheroids identifies 3D growth-specific chromatin interactions in breast cancer endocrine resistance**

Jingwei Li, Kun Fang, Lavanya Choppavarapu, Ke Yang, Yini Yang, Junbai Wang, Ruifeng Cao, Ismail Jatoi, Victor X. Jin

**Supplementary Method**

**Hi-C matrix based LOWESS normalization to remove the batch effect:** We normalized the data based on the intra-chromosomal valid pairs as only intra-chromosomal valid pairs affects the downstream analysis (e.g. TADs and SIFs). Firstly, we constructed the MD plot based on the sparse Hi-C matrix: M represents the log magnitude changes between two batches of datasets (e.g. MCF7_2D_Stein vs. MCF7_2D_merge):

$$M= {log}_{2}(\frac{{VP}_{2}}{{VP}_{1}})$$

Where ${VP}_{2}$ and ${VP}_{1}$ are valid pairs’ count in the Hi-C matrix of the first and the second dataset, respectively. D is defined as the distance between two interacting loci (the difference between X and Y coordinates in the Hi-C matrix), expressed in unit-length of the resolution of the Hi-C matrix. We then used LOWESS algorithm to find the off-set line $f_{off-set}$in the MD plot (**Fig. S4**). After fitting the data with the smoothed off-set line, we normalized the ${VP}_{2}$ with the following equation:

$${log}_{2}\left( {VP}_{2-normed} \right)= {log}_{2}\left( {VP}_{2} \right)- f_{off-set}(D)$$

Where $f_{off-set}(D)$ is the predicted value from the LOWESS regression at a distance D.

**Supplementary Figures**

**Figure S1.** Scatter plots showing the correlation of 1Mb bin-size interaction between two biological replicates for MCF10A_3D, MCF7_3D and MCF7TR_3D respectively.

In the figure, r^2^ represents correlation coefficient, and P is the corresponding P-value corrected by Bonferroni correction. A red smooth line means a perfect match (45 degree angle) between the two data points.

**Figure S2.** The PCA Plot of replicates 1 and 2 of MCF10_3D, MCF7_2D, MCF7_3D, MCF7TR_2D and MCF7TR_3D.

**Figure S3.** PCA Plots of MCF10A_2D_Stein, MCF10_3D_merge, MCF7_2D_merge, MCF7_2D_Stein, MCF7_3D_merge, MCF7TR_2D_merge and MCF7TR_3D_merge before and after LOWESS normalization.

**Figure S4.** Hi-C matrix based LOWESS normalization between MCF7_2D_Stein and MCF7_2D_merge in a chromosome-by-chromosome manner.

**Figure S5.** The comparison of TADs between MCF7TR_3D down-sampled dataset and MCF7TR_3D original dataset.


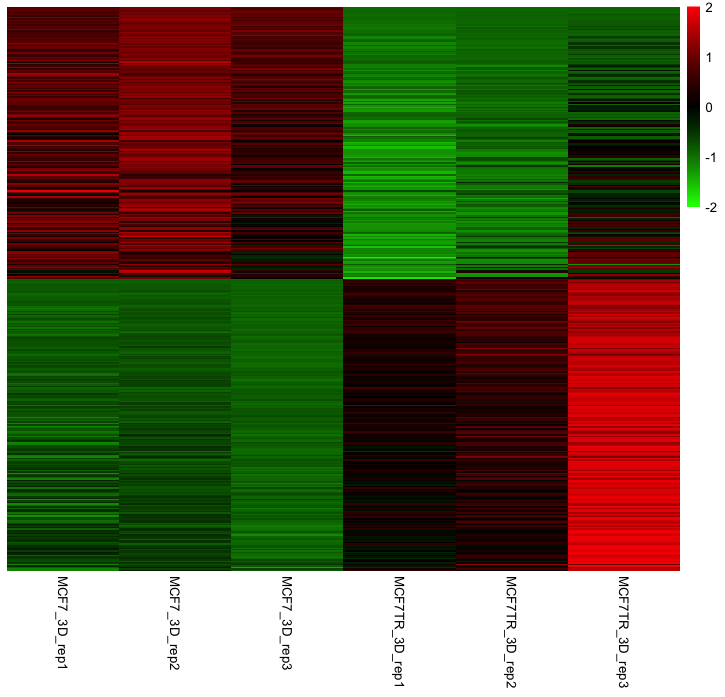
**
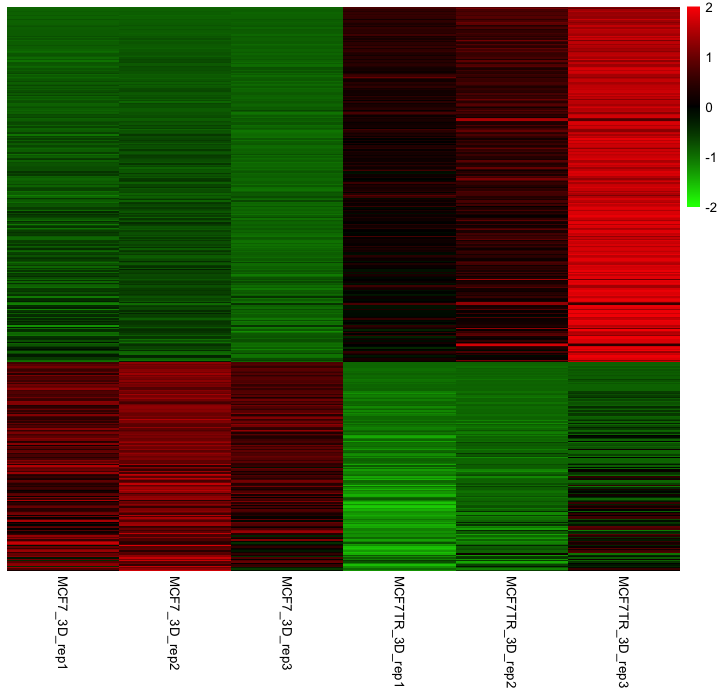
Figure S6.** Heatmaps showed that 384 of 1,678 strengthened loop genes in MCF7_3D (left panel: 185 SLG-UpR and 199 SLG-DownR) as well as 411 of 1,564 strengthened loop genes in MCF7TR_3D (right panel: 259 SLG-UpR and 152 SLG-DownR) were differentially expressed between MCF7_3D vs MCF7TR_3D, respectively.

**Figure S7.** IHC staining of ER, PR, HER2 and Ki67 in organoids of NT, PT and RT respectively with a higher magnification (40X) and a scale bar at 50 µm.

**
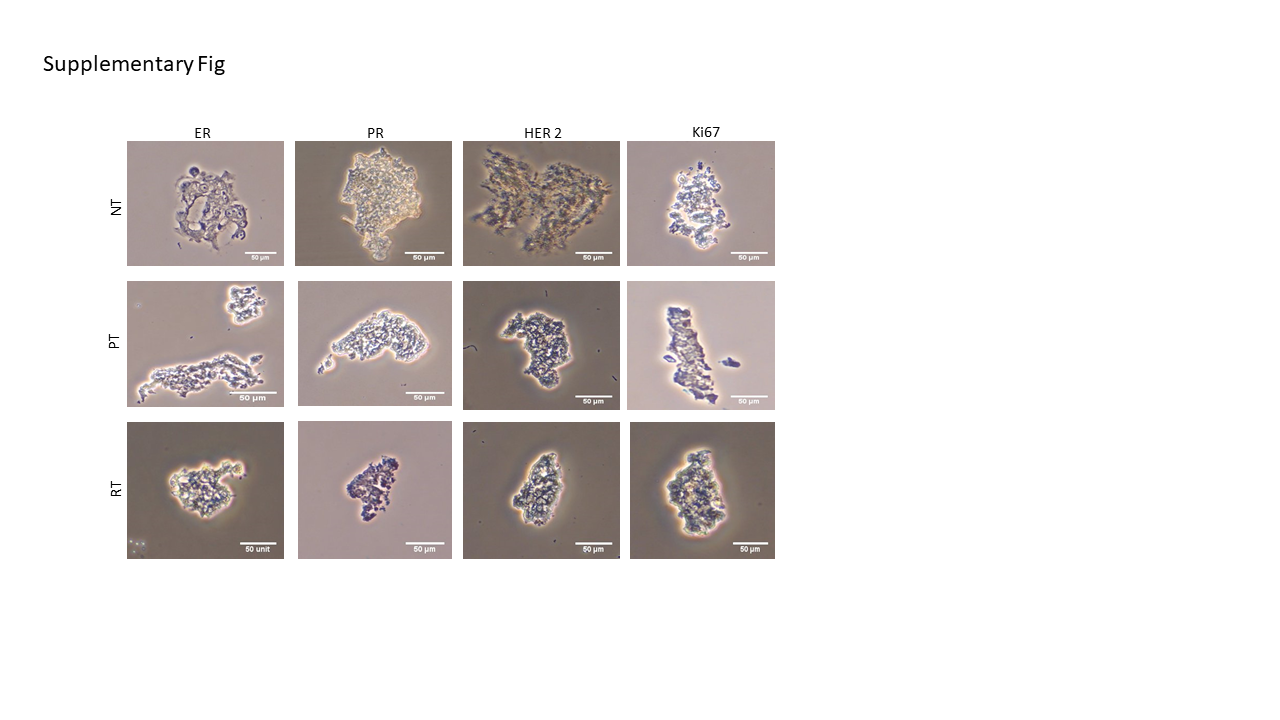
**

**Supplementary Tables**

**Table S1.** A summary of raw, uniquely mapped reads and valid pairs of Hi-C data.

| **Sample** | **Replicates** | **Raw** | **Uniquely mapped** | **Valid pairs** |
| --- | --- | --- | --- | --- |
| MCF7_3D | R1 | 243,295,349 | 152,374,220 (62.6%) | 109,316,191 |
|  | R2 | 282,082,384 | 180,134,512 (63.9%) | 120,676,998 |
| MCF7_2D | R1 | 224,108,777 | 131,740,688 (58.8%) | 93,394,187 |
|  | R2 | 201,354,069 | 119,854,597 (59.5%) | 92,847,411 |
| MCF7TR_3D | R1 | 250,035,524 | 162,674,598 (65.1%) | 112,757,125 |
|  | R2 | 95,875,862 | 63,709,944 (66.5%) | 41,741,823 |
| MCF7TR_2D | R1 | 99,201,364 | 60,967,272 (61.5%) | 16,391,645 |
|  | R2 | 91,741,610 | 50,318,300 (54.8%) | 38,727,351 |
| MCF10A_3D | R1 | 151,006,578 | 101,164,717 (67.0%) | 65,581,298 |
|  | R2 | 203,489,076 | 131,740,688 (64.5%) | 83,260,561 |
| MCF10A_2D | R1 | 289,975,071 | 135,329,068 (46.7%) | 105,746,176 |

Note: Hi-C data for MCF710A were publicly downloaded from GSE66733, for MCF7 and MCF7TR were from our previous studies under GEO accession numbers GSE108787 and GSE144380.

**Table S2.** A summary of raw and uniquely mapped reads for RNA-seq data.

| **Cell type** | **Replicates** | **Raw read counts** | **Uniquely mapped read counts** |
| --- | --- | --- | --- |
| MCF7_2D | 1 | 34,848,808 | 27,167,168 |
| MCF7_2D | 2 | 45,852,219 | 34,664,302 |
| MCF7_2D | 3 | 40,390,441 | 30,220,475 |
| MCF7TR_2D | 1 | 44,174,386 | 33,714,455 |
| MCF7TR_2D | 2 | 48,807,121 | 33,442,222 |
| MCF7TR_2D | 3 | 37,654,117 | 29,047,729 |
| MCF10A_2D | 1 | 32,941,532 | 32,386,955 |
| MCF10A_2D | 2 | 34,486,668 | 33,908,183 |
| MCF10A_2D | 3 | 40,213,691 | 39,500,846 |
| MCF7_3D | 1 | 28,708,124 | 27,653,874 |
| MCF7_3D | 2 | 29,682,546 | 28,355,012 |
| MCF7_3D | 3 | 26,601,038 | 25,388,179 |
| MCF7TR_3D | 1 | 27,887,824 | 26,989,886 |
| MCF7TR_3D | 2 | 29,397,268 | 28,445,728 |
| MCF7TR_3D | 3 | 45,897,963 | 44,472,483 |
| MCF10A_3D | 1 | 30,277,909 | 29,710,769 |
| MCF10A_3D | 2 | 300,352,63 | 29,427437 |
| MCF10A_3D | 3 | 33,329,832 | 32,659,655 |

**Table S3.** Primers for 3C-qPCR.

| **S.No** | **Name** | **Primer** |
| --- | --- | --- |
| 1 | ZDHHC7_Anchor | GTTACTGTGTACAGACATTTACTGA |
|  | ZDHHC7 | GATATTATTTGCTGAGATGATACAC |
| 2 | TEAD3_Anchor | CTTTCTTCTTGGTCTTTCTCATCTT |
|  | TEAD3 | GAGAAGTAGGAAGGCTTAGAATGTC |
| 3 | PRKD3_Anchor | CATAGAGACATCCTACAATCTATTC |
|  | PRKD3 | CTACTTTACATAGGAAAACAGAGAG |
| 4 | LATS2_Anchor | CCCGTCTCTGTTAAACATACAAAAT |
|  | LATS2 | AGAGTCTCACTCTGTCACCTAGACT |
| 5 | MET_Anchor | CTAAAGATAAAAGCTACAGACCATC |
|  | MET | GTGTATAAGTGTTCTCTTTTCTCTG |
| 6 | GAPDH_Anchor | ATGCAAGGCTTTCTCTTAAATTAGC |
|  | GAPDH | AATTCTGAGCATTCTGTAGCAAACT |

**Table S4.** Primers for RT- qPCR.

| **S.No** | **Gene** | **Sequence** | **Product** |
| --- | --- | --- | --- |
| 1 | LATS2 | 5’-GGTCACATTAAACTCACAGA-3’  5’-GACAGTTAGACACATCATCC-3’ | 139 |
| 2 | ZDHHC7 | 5’-TATAGCTCTGTCTTCAGTCC-3’  5’-GAAGATCAACAGGATTACAG-3’ | 124 |
| 3 | RASSF3 | 5’-GAGGAGATCAAAGAGAAAGT-3’  5’-GTACTTTAATGAAGCCAGTG-3’ | 103 |
| 4 | TEAD3 | 5’-CAGTACAGCTCTGCTGATAG-3’  5’-ATACTCAGTCTCCACCTTCT-3’ | 93 |
| 5 | PRKD3 | 5’-TACATGAAGGAGACCTAGTG-3’  5’-CAGAAAGTAGGAGCTTTGTA-3’ | 112 |
| 6 | ACTB | 5’-GGCATCCTCACCCTGAAGTA-3’  5’-AGGTGTGGTGCCAGATTTTC-3’ | 82 |

**Table S5.** 3D-FISH Probes for PRKD3.

|  | **Genomic region** | **Genomic Coordinates** | **RPCI name  (GRCh37/hg19)** | **Coordinates (GRCh37/hg19)** | **Size  (bp)** |
| --- | --- | --- | --- | --- | --- |
| **Fosmids** | Promoter of PRKD3 | chr2:37492865-37512865 | G248P87859G2 | chr2:37480752-37524271 | 43,520 |
|  |  |  | G248P87902C2 | chr2:37480796-37524239 | 43,444 |
|  | Distal of PRKD3 | chr2:37528136-37548136 | G248P8271H6 | chr2:37514805-37552763 | 37,959 |
|  |  |  | G248P81378D11 | chr2:37515943-37550568 | 34,626 |
